# Longitudinal strain by speckle tracking and echocardiographic parameters as predictors of adverse cardiovascular outcomes in chronic Chagas cardiomyopathy

**DOI:** 10.1007/s10554-021-02508-5

**Published:** 2022-01-13

**Authors:** Luis Eduardo Echeverría, Lyda Z. Rojas, Oscar L. Rueda-Ochoa, Sergio Alejandro Gómez-Ochoa, Miguel A. Mayer, Lisbeth Paola Becerra-Motta, Carlos Luengas, Angel M. Chaves, Jaime A. Rodríguez, Carlos A. Morillo

**Affiliations:** 1 Heart Failure and Cardiac Transplant Unit, Fundación Cardiovascular de Colombia, Calle 155A # 23-58 Urbanización El Bosque, PO. Box 681001, Floridablanca, Colombia; 2 Research Group in Cardiovascular Sciences, Research Center, Cardiovascular Foundation of Colombia, Floridablanca, Santander Colombia; 3 Research Group and Development of Nursing Knowledge (GIDCEN-FCV), Research Center, Cardiovascular Foundation of Colombia, Floridablanca, Santander Colombia; 4 Electrocardiography Research Group, Medicine School, Universidad Industrial de Santander, Bucaramanga, Colombia; 5 Research Center, Fundación Cardiovascular de Colombia, Floridablanca, Colombia; 6 Institute of Social and Preventive Medicine (ISPM), University of Bern, Bern, Switzerland; 7 Research Programme On Biomedical Informatics, Hospital del Mar Medical Research Institute, Faculty of Health and Life Sciences, Universitat Pompeu Fabra, Barcelona, Spain; 8 Department of Cardiac Sciences, Cumming School of Medicine, Libin Cardiovascular Institute, University of Calgary, Alberta, Canada; 9 Population Health Research Institute-McMaster University, Hamilton, ON Canada

**Keywords:** Echocardiography, Speckle tracking, Chagas disease, Chronic Chagas cardiomyopathy

## Abstract

To analyze the prognostic value of left ventricular global longitudinal strain (LV-GLS) and other echocardiographic parameters to predict adverse outcomes in chronic Chagas cardiomyopathy (CCM). Prospective cohort study conducted in 177 consecutive patients with different CCM stages. Transthoracic echocardiography measurements were obtained following the American Society of Echocardiography recommendations. By speckle-tracking echocardiography, LV-GLS was obtained from the apical three-chamber, apical two-chamber, and apical four-chamber views. The primary composite outcome (CO) was all-cause mortality, cardiac transplantation, and a left ventricular assist device implantation. After a median follow-up of 42.3 months (Q1 = 38.6; Q3 = 52.1), the CO incidence was 22.6% (95% CI 16.7–29.5%, n = 40). The median LV-GLS value was − 13.6% (Q1 =  − 18.6%; Q3 =  − 8.5%). LVEF, LV-GLS, and E/e′ ratio with cut-off points of 40%, − 9, and 8.1, respectively, were the best independent CO predictors. We combined these three echocardiographic markers and evaluated the risk of CO according to the number of altered parameters, finding a significant increase in the risk across the groups. While in the group of patients in which all these three parameters were normal, only 3.2% had the CO; those with all three abnormal parameters had an incidence of 60%. We observed a potential incremental prognostic value of LV-GLS in the multivariate model of LVEF and E/e′ ratio, as the AUC increased slightly from 0.76 to 0.79, nevertheless, this difference was not statistically significant (p = 0.066). LV-GLS is an important predictor of adverse cardiovascular events in CCM, providing a potential incremental prognostic value to LVEF and E/e′ ratio when analyzed using optimal cut-off points, highlighting the potential utility of multimodal echocardiographic tools for predicting adverse outcomes in CCM.

## Introduction

Chagas disease (CD), caused by the parasite *Trypanosoma cruzi* (*T. cruzi*), is a neglected disease endemic in Latin America that has spread worldwide due to increased migration from endemic countries, becoming a global public health threat [1]. CD is a significant cause of heart failure (HF) and mortality in endemic countries, as almost half of all seropositive *T. cruzi* individuals will develop the cardiac form of the disease, known as chronic Chagas cardiomyopathy (CCM), within one to three decades after the initial infection [2]. CCM has usual and pathognomonic cardiac manifestations with different pathophysiological pathways and is characterized by a persistent myocardial inflammatory response, leading to progressive and diffuse cardiac fibrosis [3]. Fibrosis leads to a reduction in ejection fraction (EF) and consequent HF due to focal scarring with a predilection for the cardiac conduction system, promoting a severe arrhythmogenic condition [3]. These lesions lead to severe brady and tachyarrhythmias at higher prevalence than other cardiomyopathies [4, 5]. Moreover, the myocardium's replacement by fibrotic infiltration facilitates the development of ventricular aneurysms, which serve as areas for mural thrombus development [6]. The leading causes of death in CCM patients are sudden death (related to life-threatening arrhythmias) and HF [7, 8]. In this context, echocardiography plays a crucial role, as it provides information regarding systolic dysfunction, left ventricle (LV) mural thrombus formation, and the presence and location of scars and aneurysms [9].

Multiple clinical studies have documented the predictive value of echocardiographic parameters on outcome in several cardiomyopathies, including LVEF, left atrial (LA) volume, E/e′ ratio, among others [10–12]. In this context, several studies have reported the excellent performance of LV-GLS in detecting early myocardial abnormalities and predicting new-onset arrhythmias in CCM, even in patients with normal ECG without abnormalities on 2D echocardiography [10, 13–16]. However, the prognostic value of abnormal LV-GLS has not yet been robustly assessed [16–18]. Thus, this study's objective is to evaluate the prognostic value of LV-GLS, added to conventional echocardiographic parameters, in predicting adverse cardiovascular outcomes in CCM, such as mortality, heart transplantation (HT), and left ventricle assist device (LVAD) implantation. We hypothesize that the LV-GLS will have a significant incremental prognostic value for predicting the composite outcome in this population.

## Methods

### Study population and screening

A prospective cohort study was conducted between 2015 and 2020 in the Heart Failure service at the Cardiovascular Foundation of Colombia, in Floridablanca, Colombia. All patients were recruited during their medical appointments at the institution, which is located in a Chagas disease-endemic area.

Inclusion criteria were as follows: (a) > 18 years old, (b) positive IgG antibodies for *T. cruzi*, and (c) echocardiographic (Echo) or electrocardiogram (ECG) abnormalities consistent with CCM (i.e. left anterior fascicular block, right bundle branch block, atrioventricular blocks, ventricular premature complexes, atrial fibrillation or flutter, bradycardia ≤ 50 beats/min, or echocardiographic findings suggestive of myocardial impairment) as evaluated by a cardiologist. Patients with uncontrolled hypertension, history of coronary heart disease, and mitral stenosis were excluded. Patients were classified by severity stages of CCM based on symptoms, ECG, and Echo results, according to the recommendations of the American Heart Association (AHA); Stage B (ECG abnormalities consistent with CCM without signs or symptoms of heart failure regardless of their global ventricular function), Stage C (ECG abnormalities consistent with CCM, LVEF < 55% and current or previous symptoms of HF), and Stage D (ECG abnormalities consistent with CCM and LVEF < 55% and refractory symptoms of HF at rest despite optimized clinical treatment). A cardiologist with expertise in CCM performed this classification.

At the time of enrollment, all patients underwent a structured interview, evaluating sociodemographic features, past medical history and clinical characteristics, and physical examination. This study complied with the Declaration of Helsinki for clinical studies and the Resolution 008430 of October 4, 1993, of the Ministry of Health of Colombia, which establishes the ethical principles, scientific, technical and administrative standards for health research in the country. All patients provided their written informed consent, and the institutional Research Ethics committee approved the protocol.

### Echocardiography methods

Transthoracic echocardiography was performed using a GE Vivid S6 ultrasound system with an M4S matrix-array transducer of 1.6–4.3 MHz. Acquisitions were performed by a single certified and experienced cardiac sonographer blinded to the patient data. All echocardiograms were read and measured by a single cardiologist certified in echocardiography. Cardiac dimensions and Doppler measurements were obtained following the American Society of Echocardiography and The European Association of Echocardiography recommendations [19]. M-mode echocardiography was used to measure left atrial (LA) diameter and LV end-diastolic and end-systolic diameters (as recommended by the 2010 Guidelines of the American Society of Echocardiography). Two-dimensional LA and LV volumes were determined using modified Simpson's rule, with images obtained from the apical four-chamber and two-chamber views. Pulsed-wave Doppler was performed in the apical four-chamber view. From transmitral recordings, early peak (E) and late (A) diastolic filling velocities, E/A ratio, E-wave deceleration time, velocity–time integral (VTI) of the E-wave (VTIE), A wave VTI (VTIA), and LA filling fraction [VTIA/(VTIE + VTIA)] were obtained. The e′ lateral velocity (tissue Doppler) and the E/e′ ratio were calculated. Isovolumic relaxation time was measured from continuous-wave Doppler obtained in the apical five-chamber view. RV systolic pressure was derived from continuous-wave Doppler interrogation of tricuspid regurgitation. RV systolic function was evaluated by measuring the peak systolic myocardial velocity (RV S0) of the lateral tricuspid annulus and tricuspid annular plane systolic excursion (TAPSE). The longitudinal strain was obtained from the apical three-chamber, apical two-chamber, and apical four-chamber views by speckle-tracking echocardiography. The software automatically defined the region of interest (ROI) for the entire myocardial layer divided into six color-coded segments. Special attention was given not to include pericardium or myocardial trabeculae, and in most cases, a manual correction was required. Three cardiac cycles from each view were recorded for online analyses with a frame rate > 50 frames per second. Peak negative longitudinal strain was assessed in 16 LV segments, defined as the peak negative value during the entire cardiac cycle, including post systolic shortening, and was averaged as LV-GLS. LV-GLS analyses were not feasible in fourteen patients due to persistently poor tracking.

### Outcomes definition

The primary outcome was a composite endpoint of death, left ventricular assist (LVAD) device implantation, or cardiac transplantation. Follow-up data were obtained during a clinical follow-up appointment, clinical records review, and telephone interviews following a standardized protocol. Investigators contacted the patients once a month during the first 6 months after the initial evaluation and then contacted by phone every 6 months.

### Statistical analysis

Categorical variables are presented as total numbers and proportions. The normality of continuous variables was assessed by histograms and the Shapiro–Wilk test. Variables with normal distribution are presented as mean ± standard deviation, otherwise as median with first and third quartiles. Survival analyses were performed using the Kaplan–Meier method, life table, and Cox proportional-hazards models. To identify the variables that were independently predictive of mortality, univariate and multivariate analysis using Cox's proportional regression model was performed. After identifying the variables potentially associated with the composite outcome in the univariate models, we constructed a multivariate model including all predictors with a significance threshold of p < 0.1. Finally, we used a backward selection method to exclude sequentially variables based on both the statistical significance (using the p-value) and its contribution to the model (based on the R2). We also assessed the model fit using the Akaike information criterion (AIC) and Bayesian Information Criteria (BIC) parameters. We quantified the echocardiographic variables' predictive ability with Harrell's C statistic and the area under the receiver operating characteristic curve (AUC-ROC). The Youden index was used to identify the best cut-off for Echo variables to predict adverse cardiovascular outcomes. A p-value < 0.05 was considered significant. All statistical tests were two-sided. All data were analyzed using STATA Statistical Software version 15.0.

## Results

One hundred seventy-seven consecutive patients with different stages of Chagas disease were included (55.4% males; median age 61 years, Q1: 53 to Q3: 67 years). Among them, 50 patients were classified as stage B, 55 as stage C, and 72 as stage D. Clinical characteristics are summarized in Table 1. Most of the patients (52%) were in NYHA class I; 132 patients (75%) were receiving beta-blockers, 123 (69%) angiotensin receptor blockers or angiotensin-converting enzyme inhibitors, 70 (39%) diuretics, and 59 (33%) oral anticoagulants.

**Table 1:** Baseline characteristics of the patients with chronic CCM and predictors of the composite outcome (n = 177)

Variables	Patients without CO events (n = 137)	Patients with CO events (n = 40)	HR (95% CI)	p-Value
Age (years)	59 (51; 65)	66 (58; 74)	1.04 (1.01–1.08)	**0.006**
Males	78 (56.93)	20 (50.00)	0.81 (0.44–1.51)	0.516
BMI (kg/m^2^)	26.37 (23.33; 29.33)	22.24 (19.89; 25.14)	0.81 (0.74–0.89)	**< 0.001**
Obesity	35 (25.55)	1 (2.50)	0.084 (0.01–0.61)	**0.015**
Hypertension	66 (48.18)	23 (57.50)	1.35 (0.72–2.53)	0.299
Smoking	3 (3.53)	0 (0)	–	–
NYHA				
I–II	127 (92.70)	25 (62.50)	Reference	
III–IV	10 (7.30)	15 (37.50)	5.09 (2.68–9.69)	**< 0.001**
ACEI	41 (29.93)	24 (60.00)	2.76 (1.47–5.21)	**0.002**
ARB	48 (35.04)	12 (30.00)	0.81 (0.41–1.59)	0.541
Beta-blockers	93 (67.88)	39 (97.50)	14.69 (2.02–106.94)	**0.008**
Aldosterone antagonists	60 (43.80)	32 (80.00)	4.01 (1.85–8.71)	**< 0.001**
Diuretics	40 (29.20)	30 (75.00)	5.52 (2.70–11.30)	**< 0.001**
Digitalis	14 (10.22)	12 (30.00)	2.87 (1.46–5.66)	**0.002**
Ivabradine	1 (0.73)	2 (5.00)	3.24 (0.78–13.47)	0.105
Antiplatelet agents	38 (27.74)	8 (20.00)	0.69 (0.32–1.50)	0.352
Oral anticoagulants	39 (28.47)	20 (50.00)	2.10 (1.13–3.91)	**0.019**

This table contains % for categorical variables and median (first and third quartile) for continuous variables. The p-values were calculated based on the hazard ratio and its 95% confidence interval

*BMI* Body Mass Index, *NYHA* New York Heart Association Scale, *ACEI* angiotensin-converting enzyme inhibitor, *ARB* angiotensin receptor blocker, *LVEF* left ventricle ejection fraction, *LV-GLS* global longitudinal strain

Bold values indicate a* p*-value < 0.05

### Composite outcome

After a median follow-up of 42.3 months (Q1 = 38.6; Q3 = 52.1), the CO event rate was 23% (95% CI 16.4% to 28.8%, n = 40), for a CO event rate of 0.18 per 1000 person-years (95% CI 0.09–0.23). Among these, 34 patients died, five patients underwent heart transplant (HT), and one underwent left ventricular assist device (LVAD) implantation during the follow-up period.

Baseline echocardiographic characteristics of the included patients are summarized in Table 2. The median LVEF value was 44% (Q = 31; Q3 = 56). Patients in NYHA functional class III and IV had a lower LVEF value than the patients in class I and II (median LVEF: 27% vs. 47%, respectively; p = 0.001). Moreover, LVEF was correlated with multiple relevant echocardiographic variables, including the E/e′ ratio (r =  − 0.41, p < 0.001), LV mass index (r =  − 0.65, p < 0.001), and TAPSE (r = 0.60, p < 0.001). Most of the echocardiographic parameters were significantly associated with the CO, except for the presence of left LV aneurysms (Table 2).

**Table 2:** Baseline echocardiographic characteristics of the patients and predictors of the composite outcome in chronic Chagas cardiomyopathy

Variables	Patients without CO events (n = 137)		Patients with CO events (n = 40)	HR (95% CI)	p-Value
LVEF (%)		50 (38; 59)	28 (19; 36)	0.92 (0.89–0.94)	**< 0.001**
LVEF (%)					
≥ 40		98 (71.53)	7 (17.50)	Reference	
**< **40		39 (28.47)	33 (82.50)	8.75 (3.86–19.81)	**< 0.001**
End-diastolic volume, ml		60 (47; 83)	98 (69; 151)	1.02 (1.01–1.02)	**< 0.001**
End-systolic volume, ml		32 (21; 50)	68 (44; 119)	1.02 (1.01–1.03)	**< 0.001**
LV Mass Index		106 (83; 133)	151 (117; 185)	1.01 (1.01–1.02)	**< 0.001**
LV aneurysms		23 (16.79)	10 (26.32)	1.46 (0.71–3.00)	0.308
LA Volume Index, ml/m^2^	39 (29; 52)		67 (53; 88)	1.02 (1.01–1.03)	**< 0.001**
Lateral e, cm/s	9 (6; 12)		6 (5; 9)	0.83 (0.74–0.94)	**0.003**
Lateral a, cm/s	10 (8; 13)		7 (5; 10)	0.75 (0.67–0.84)	**< 0.001**
E/e′ ratio	7.35 (5; 9)		10.05 (8; 14)	1.16 (1.08–1.23)	**< 0.001**
TAPSE	18 (15; 21)		12 (10; 15)	0.81 (0.75–0.87)	**< 0.001**
MAPSE	10 (8; 13)		7 (5; 9)	0.81 (0.73–0.89)	**< 0.001**
PASP	30 (26; 34)		38 (31; 50)	1.07 (1.05–1.10)	**< 0.001**

This table contains % for categorical variables and median (first and third quartile) for continuous variables

*LVEF* left ventricle ejection fraction, *PSAP* pulmonary artery systolic pressure

Bold values indicate a* p*-value < 0.05

### Speckle tracking and composite outcome

**Fig. 1:**
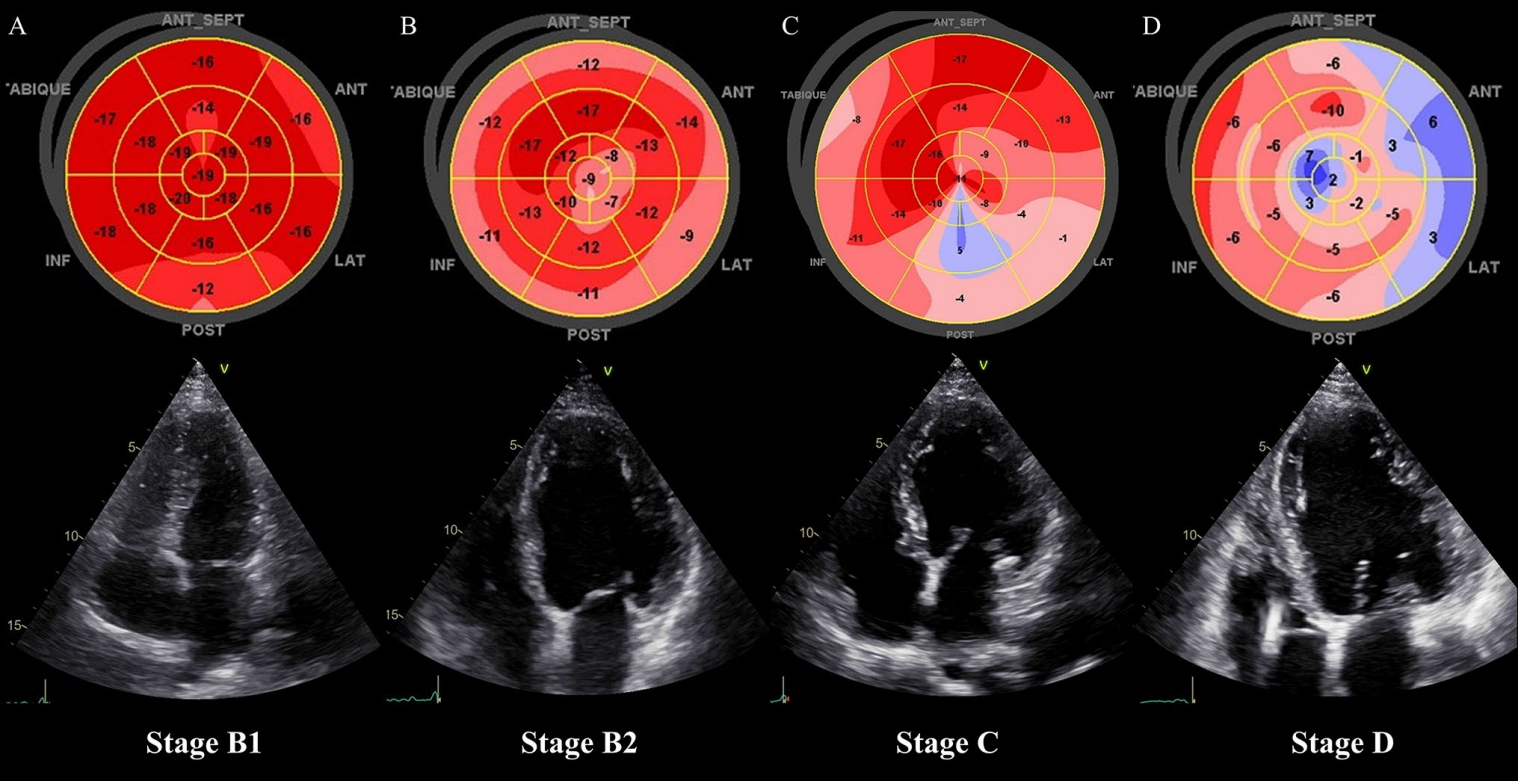
The progression of the global longitudinal strain of the left ventricle in patients with Chronic Chagas Cardiomyopathy. **A** Stage B1 (structural cardiomyopathy without global ventricular function involvement); **B** Stage B2 (structural cardiomyopathy with a mildly deteriorated global ventricular function); **C** Stage C (symptomatic heart failure with moderate to severe deteriorated global ventricular function); **D** Stage D (refractory heart failure)

**Fig. 2:**
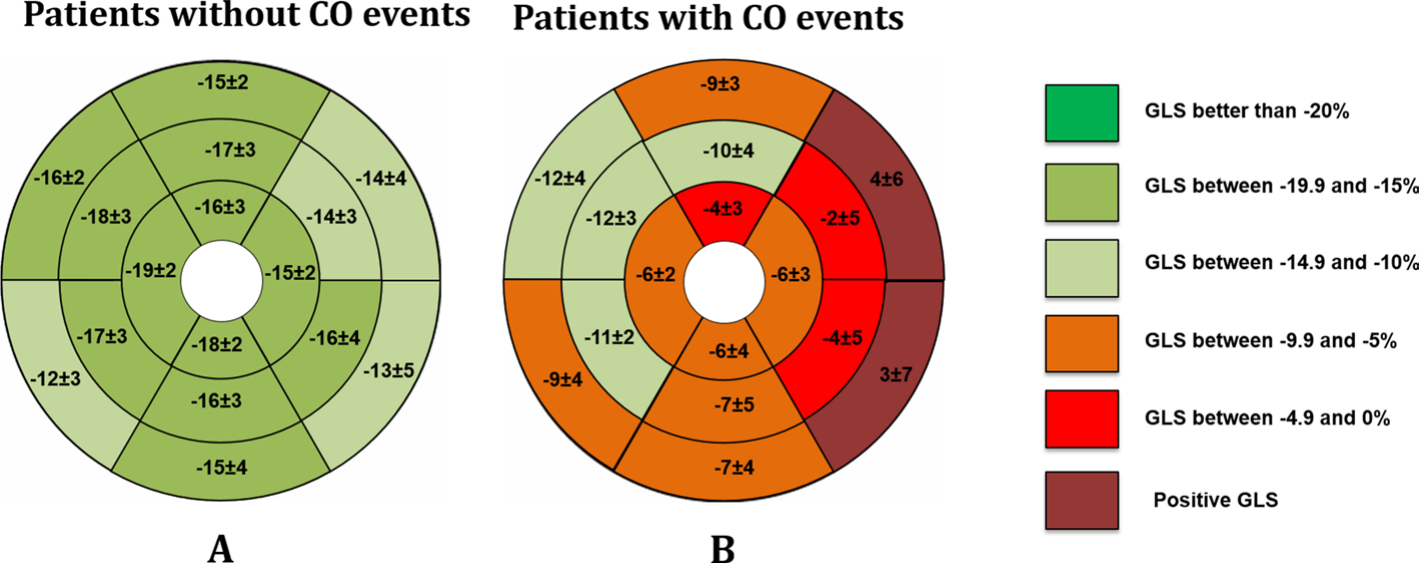
Bull' s-eye plot of the left ventricle. Segmental longitudinal strain values with three concentric circles representing apex (inner circle), mid (middle circle), and base (outer circle). **A** Average LV-GLS in patients without the composite outcome. **B** Average of LV-GLS in patients with the composite outcome

The median global longitudinal strain (LV-GLS) value was − 13.6 (Q1 =  − 18.6; Q3 =  − 8.5) and was significantly worse in patients with reduced LVEF when compared to patients with preserved LVEF (median: − 9.7 vs. − 19.3, respectively; p < 0.001) (Fig. 1). As expected, a strong inverse correlation between LVEF and LV-GLS was observed (r =  − 0.91; p < 0.001). Table 3 summarizes the findings of segmental speckle tracking strain in patients and the CO incidence. LV-GLS of the individuals with the CO was significantly worse (median: − 7.7 vs. − 15.5, compared with patients without the CO; p < 0.001), being the basal anterolateral (median: 4; Q1: − 3, Q3: 6), basal inferolateral (median: 3; Q1: − 6, Q3: 5) and mid anterolateral segments (median: − 2; Q1: − 8, Q3: 4) the most abnormal in the individuals with the CO (Fig. 2). In addition, almost all speckle tracking segment values were significantly associated with the CO (Table 3).

**Table 3:** Baseline ventricular longitudinal speckle tracking strain characteristics as predictors of the composite outcome in chronic Chagas cardiomyopathy

Variables	Patients without CO events (n = 137)	Patients with CO events (n = 40)	HR (95% CI)	p-Value
LV LV-GLS	− 15.5 (− 19.1; − 10.4)	− 7.7 (− 11.2; − 4.9)	1.23 (1.14; 1.32)	**< 0.001**
Apical septal	− 19 (− 24; − 10)	− 6 (− 19; 3)	1.05 (1.03; 1.08)	**< 0.001**
Apical lateral	− 15 (− 21; − 8)	− 6 (− 13; 2)	1.05 (1.02− 1.08)	**< 0.001**
Apical inferior	− 18 (− 22; − 11)	− 6 (− 13; 3)	1.05 (1.03; 1.08)	**< 0.001**
Apical anterior	− 16 (− 21; − 7)	− 4 (− 9; 0)	1.08 (1.04; 1.12)	**< 0.001**
Mid anteroseptal	− 18 (− 22; − 12)	− 12 (− 17; − 8)	1.06 (1.02–1.10)	**0.003**
Mid anterior	− 17 (− 21; − 12)	− 10 (− 14; − 6)	1.09 (1.05–1.14)	**< 0.001**
Mid anterolateral	− 14 (− 21; − 6)	− 2 (− 8; 4)	1.09 (1.05–1.13)	**< 0.001**
Mid inferolateral	− 16 (− 21; − 7)	− 4 (− 10; 4)	1.08 (1.04–1.11)	**< 0.001**
Mid inferior	− 16 (− 20; − 11)	− 7 (− 12; − 5)	1.08 (1.04–1.12)	**< 0.001**
Mid inferoseptal	− 17 (− 20; − 12)	− 11 (− 14; − 5)	1.09 (1.05–1.15)	**< 0.001**
Basal anteroseptal	− 16 (− 19; − 11)	− 12 (− 15; − 8)	1.03 (0.99–1.07)	0.109
Basal anterior	− 15 (− 18; − 9)	− 9 (− 12; − 1)	1.07 (1.03–1.11)	**< 0.001**
Basal anterolateral	− 14 (− 18; − 3)	4 (− 7; 8)	1.09 (1.05–1.12)	**< 0.001**
Basal inferolateral	− 13 (− 17; 3)	3 (− 10; 6)	1.05 (1.02–1.08)	**< 0.001**
Basal inferior	− 15 (− 19; − 10)	− 7 (− 13; 0)	1.08 (1.04–1.12)	**< 0.001**
Basal inferoseptal	− 12 (− 15; − 8)	− 9 (− 11; − 5)	1.09 (1.04–1.13)	**< 0.001**

*LV-GLS* global longitudinal strain

Bold values indicate a* p*-value < 0.05

**Fig. 3:**
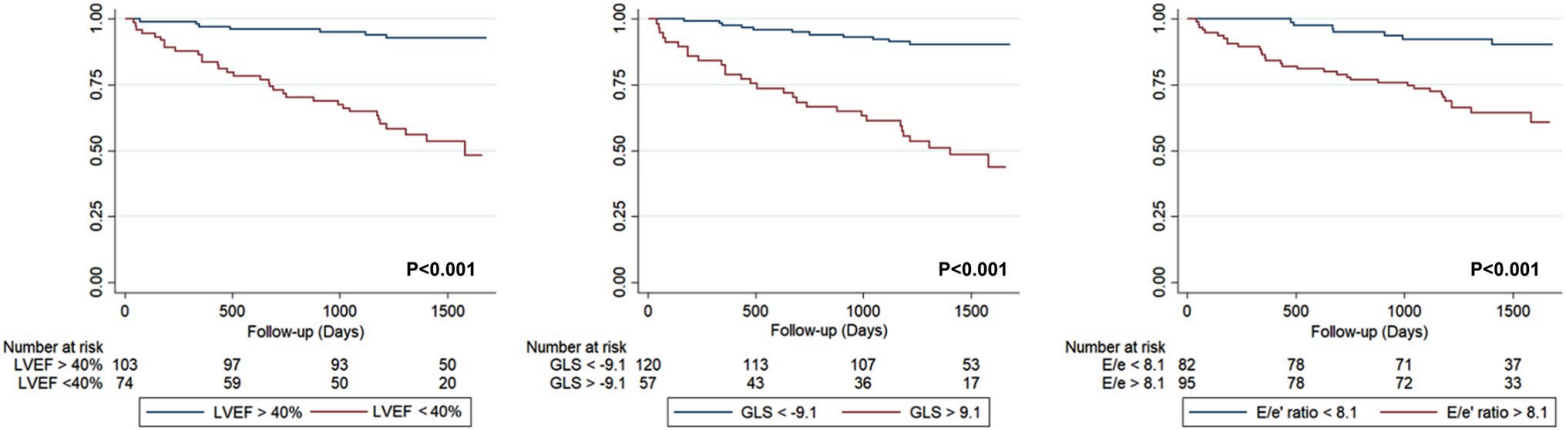
Kaplan–Meier curve for the composite outcome incidence for **a** LVEF, **b** LV-GLS, and **c** E/e′ ratio

**Fig. 4:**
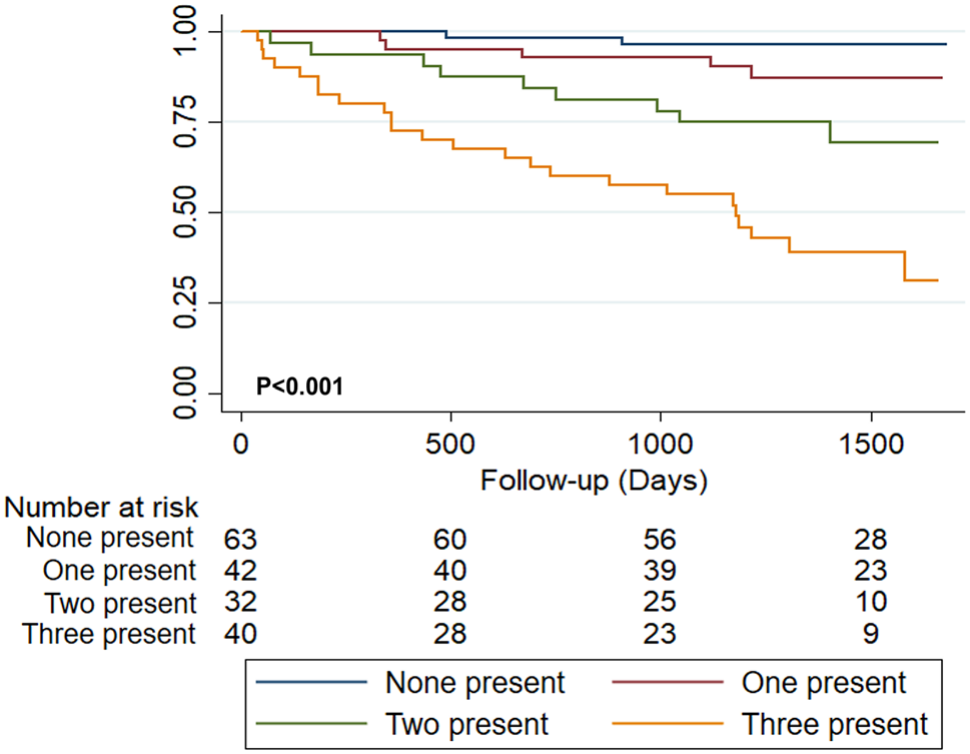
Kaplan–Meier curve for the composite outcome by number of altered independent echocardiographic predictive parameters (LVEF, LV-GLS, and E/e′ ratio)

**Fig. 5:**
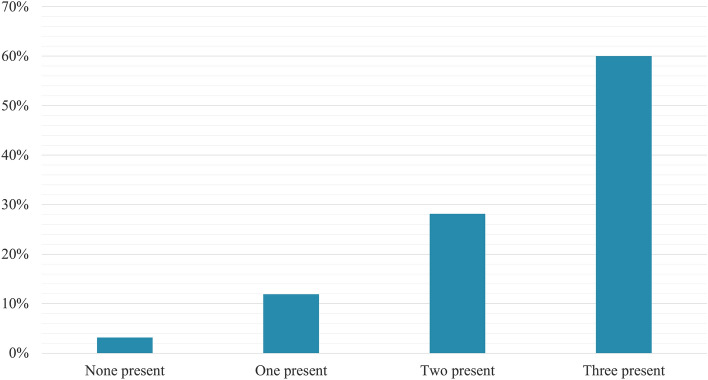
Raw incidence proportion of the composite outcome of mortality, heart transplantation and LVAD implantation according to the number of altered echocardiographic prognostic markers (LVEF < 40%, LV-GLS >  − 9%, and E/e′ ratio > 8.1) in chronic Chagas cardiomyopathy

Multivariate Cox proportional-hazards analysis identified LVEF (HR 0.95; 95% CI 0.923–0.982; p = 0.002), left atrial volume index (HR 1.01; 95% CI 1.01–1.02; p = 0.014) and TAPSE (HR 0.89; 95% CI 0.82–0.98; p = 0.020) as independent continuous markers for predicting the CO. We aimed to analyze cut-off points that may be clinically useful for the variables significantly associated with the composite outcome in the bivariate analysis. After the analysis was performed using dichotomic echocardiographic variables, we observed that a model including LVEF, LV-GLS, and E/e′ ratio with cut-off points of 40%, − 9, and 8.1, respectively, had the better fit for the prediction of the composite outcome (AIC: 261.807, BIC: 265.919), also suggesting that these variables were independent predictors of the CO (Table 4 and Fig. 3). Finally, we combined these three echocardiographic markers and evaluated the CO's risk according to the number of abnormal parameters, finding a significant increase in the risk across the groups (Fig. 4). In the group of patients in which all these three parameters were below the cut-off value, the CO's event rate was only 3.17% (95% CI 0.78–12.04%). In contrast, those in which all these parameters were abnormal had a CO event rate of 60% (95% CI 45.31–75.55%), primarily driven by death (Fig. 5). A potential incremental prognostic value of LV-GLS in the multivariate model of LVEF and E/e′ ratio was observed, as the AUC increased slightly with the inclusion of LV-GLS, from 0.76 to 0.79. Nevertheless, this difference was not statistically significant (p = 0.066).

**Table 4:** Multivariable Cox proportional-hazards analysis for predicting the composite outcome in patients with chronic Chagas cardiomyopathy

Variables	HR	95% CI	p-Value
LVEF (< 40% vs ≥ 40%)	3.15	1.15–8.64	**0.025**
LV-GLS (> − 9 vs < − 9)	2.82	1.21–6.54	**0.016**
E/e′ ratio (> 8.1 vs < 8.1)	2.48	1.06–5.78	**0.034**

Bold values indicate a* p*-value < 0.05

## Discussion

In this cohort study, we evaluated the role of LV speckle tracking strain in predicting adverse cardiovascular outcomes in a cohort of one hundred seventy-seven patients with chronic Chagas cardiomyopathy, mainly in the stage D of the disease (40.68%). We observed a significant incremental prognostic value of LV speckle tracking strain predicting a CO of mortality, heart transplantation (HT), and LVAD implantation. LV-GLS also increased the AUC-ROC of a model including LVEF and E/e′ ratio, contributing to the predictive value of previously established echocardiographic measures of prognosis in CCM. Interestingly, we observed that in patients with these three parameters over their selected cut-off values, the composite outcome rate was around 60% despite optimal medical therapy for their heart failure, contrary to a CO rate below 4% in those without these predictive parameters present. Therefore, the analysis of the role of these echocardiographic measures in the course of CCM acquires a significant relevance considering the necessity of improving our ability to identify these high-risk individuals as early as possible, allowing a close follow-up and a timely optimization of the therapeutic strategies offered for this particular group of patients.

The assessment of myocardial strain by speckle tracking represents nowadays a useful and cost-effective tool for the evaluation of the cardiac function, with relevant implications in risk stratification and the prognosis evaluation in patients with cardiovascular diseases. For example, the subepicardial longitudinal strain has been identified as an independent risk factor for adverse cardiovascular events in patients with hypertension and hypertensive heart disease, even after adjusting by other relevant clinical and echocardiographic parameters [20, 21]. Similarly, the study of Liu et al., performed in patients with type 2 diabetes mellitus, suggested that GLS had a significant incremental prognostic value for predicting adverse cardiovascular outcomes in a model including clinical data, HbA1c, and other echocardiographic variables [22]. Furthermore, strain by speckle tracking has shown to significantly improve the prognostic assessment of patients with coronary artery disease, valvular heart disease, atrial fibrillation, hypertrophic cardiomyopathy, and heart failure, both in the setting of reduced and preserved ejection fraction [23–29]. Nevertheless, there is scarce evidence regarding its role in an infectious cardiomyopathy such as CCM.

At first, speckle tracking strain has the capacity of identifying subclinical myocardial dysfunction even in individuals with CD without symptoms, ECG, or conventional echocardiographic abnormalities (the so-called indeterminate form of the disease) [30]. Multiple studies have found that patients in the indeterminate form may show incipient cardiac damage documented by abnormal longitudinal and radial strain of the LV compared to healthy controls [16, 31]. Moreover, radial, circumferential, and longitudinal LV speckle-tracking strain values have been correlated with disease progression and the degree of myocardial fibrosis as measured by cardiac magnetic resonance imaging, highlighting the utility of this diagnostic method in CD and CCM [30, 32]. In addition, speckle tracking strain has been shown to have clinical relevance in the prognostic assessment of patients with heart failure independently of LVEF. *Kalam *et al*.* performed a meta-analysis comprising 16 cohort studies, observing that each SD change in the absolute value of baseline LV-GLS was independently associated with mortality (HR 0.50 95% CI 0.36 to 0.69; p < 0.002); this association was stronger than LVEF, as the hazard ratio (HR) per SD variation in LV-GLS was associated with a reduction in mortality 1.62 (95% CI 1.13 to 2.33; p = 0.009) times greater than the HR per SD change in LVEF [33]. Furthermore, LV-GLS could be even more useful in predicting adverse outcomes in the context of HF with preserved ejection fraction (HFpEF), as suggested by the study of Morris et al., a systematic review and meta-analysis of 22 studies that highlighted the significantly lower value of LV-GLS in individuals with HFpEF compared to asymptomatic patients and healthy subjects. Furthermore, two studies included in this meta-analysis revealed that patients with an abnormal LV-GLS had a significantly higher risk of CV mortality (HR 2.14; 95% CI 1.26–3.66) and heart failure hospitalization (HR 1.94; 95% CI 1.22–3.07) even after adjusting by clinical and echocardiographic confounders [34]*.*

Despite this, only one study has previously evaluated the speckle tracking strain technique in the context of CCM as a predictor of adverse outcomes. In this study, Santos et al. analyzed 112 patients with dilated cardiomyopathy (81 with CCM and 31 with idiopathic dilated cardiomyopathy), observing that after a median follow-up of 18.2 months, LV-GLS was an independent predictor of adverse events. The patients with LV-GLS >  − 12% had a higher risk of the composite outcome of death, heart failure hospitalization, or need for heart transplantation. The predictive value was incremental to LVEF, and E/e′ ratio (HR 1.463, 95% CI 1.130–1.894; p = 0.004), as observed in the present study [18].

Nevertheless, the observation of an additive value of LV-GLS highlights the need to consider other standard echocardiographic measures such as LVEF and E/e′ ratio, assessing both systolic and diastolic dysfunction, respectively, in the integral assessment of CCM patients [9.11]. Furthermore, additional echocardiographic measures have been evaluated as predictor tools for assessing the prognosis of these patients. For example, a recently published substudy of the BENEFIT trial evaluated the value of LV wall motion score index (WMSI) in predicting a composite outcome of death, resuscitated cardiac arrest, insertion of a pacemaker or an implantable cardioverter-defibrillator, sustained ventricular tachycardia, cardiac transplantation, new heart failure, stroke or transient ischemic attack, or an embolic event in CCM [35]. The researchers of this study observed a significant graded increase in the risk of the composite outcome with worsening WMSI, having patients with a LV WMSI > 1.5 a double risk of these adverse outcomes compared to the ones with a LV WMSI = 1 (HR 2.15; 95% CI 1.24–3.74) [35].

Our findings have relevant clinical implications for patients across all stages of CCM. The observation of a potentially independent association of GLS with a composite outcome consisting of death, LVAD implantation, and HT highlights the utility of incorporating this measure systematically into the risk evaluation of patients with CCM. Furthermore, the speckle tracking strain method has several technical and economic advantages over other complementary imaging strategies, such as computed tomography and magnetic resonance; therefore, considering that fibrosis represents the hallmark of myocardial involvement in CD, speckle tracking strain may significantly optimize the imaging assessment of these patients [36–38]. Moreover, the observation of an important role of GLS in the prognosis of CCM should prompt the analysis of its value for differentiating patients with the indeterminate form of the disease from those with early myocardial involvement, which are usually misclassified due to sub-optimal criteria [39]. Finally, additional studies are required for evaluating the potential added value of speckle tracking strain in the evaluation of progression risk from the indeterminate form to the cardiac clinical form, favoring early interventions such as anti-parasite treatment and a closer clinical follow-up in high-risk patients [13, 18].

### Study limitations

The main limitation of the present study is the small sample analyzed, as it precluded a stratified analysis and the subsequent creation of a clinical score due to the lack of a validation cohort. Notably, although the recent Guideline for Multimodality Cardiac Imaging in Patients with Chagas disease supports the use of 2D echocardiography to calculate the LVEF through the biplane method of disks (the Simpson rule), we are aware that this technique may be limited by the presence of apical aneurysms in the left ventricles [40]. Acquisition and analyses were performed by a single individual, limiting reproducibility and preventing us from determining intra-observer variability. Furthermore, we did not have any data on the heart rate of the evaluated patients nor information regarding the presence of atrial fibrillation during the echocardiogram, limiting the possibility of considering these relevant variables in the acquisition of the strain measures. On the other hand, the sample size and the follow-up were not pre-specified, as the original goal of this cohort was to perform a cross-sectional analysis of the patients with CD across the different stages of the disease. Moreover, no sensitivity analysis assessing a survival endpoint of cardiovascular death was performed; however, most of the outcome events represented this endpoint, which suggests a similar trend compared to the result observed for the CO. Finally, we did not measure radial and circumferential strain, two methods that may have given valuable information in assessing CCM prognosis.

## Conclusions

In the present study, LV-GLS was a significant predictor of adverse cardiovascular events in CCM patients, providing a potential incremental prognostic value to LVEF and E/e′ ratio when analyzed using optimal cut-off points. The findings of this study have important clinical implications, highlighting the utility of a multimodal biomarker approach in facilitating the identification of patients who may benefit from earlier initiation of pharmacological therapy to delay the disease's progression. The utility of this tool for predicting adverse outcomes in CCM needs to be validated in larger cohorts, including radial and circumferential strain measurements.

## Abbreviations

AUC-ROC: Area under the receiver operating characteristic curve
BMI: Body mass index
CCM: Chronic Chagas cardiomyopathy
ECG: Electrocardiogram
Echo: Echocardiogram/echocardiographic
HF: Heart failure
HR: Hazard ratio
LV-GLS: Left ventricular global longitudinal strain
LA: Left atrial
LV: Left ventricular
LVEF: Left ventricular ejection fraction
NT-proBNP: NT-terminal pro B-type natriuretic peptide
NYHA: New York Heart Association
RV: Right ventricular
